# Anticancer Activity of *Delphinium semibarbatum* Alkaloid Fractions against LNCaP, and DU 145 Human Prostate Cancer Cells through the Intrinsic Apoptotic Pathway

**DOI:** 10.22037/ijpr.2021.115462.15382

**Published:** 2021

**Authors:** Mohammadreza Lotfaliani, Mustafa Ghanadian, Seyed Abdulmajid Ayatollahi, Mahmoud Aghaei, Farzad Kobarfard

**Affiliations:** a *Department of Pharmacognosy, School of Pharmacy, Shahid Beheshti University of Medical Science, Tehran, Iran.*; b *Department of Pharmacognosy, School of Pharmacy, Shaheed Sadoughi University of Medical Science and Health Service, Yazd, Iran. *; c *Department of Pharmacognosy, Isfahan Pharmaceutical Sciences Research Center, School of Pharmacy, Isfahan University of Medical Sciences, Isfahan, Iran. *; d *Phytochemistry Research Center, Shahid Beheshti University of Medical Science, Tehran, Iran. *; e *Department of Clinical Biochemistry, School of Pharmacy, Isfahan University of Medical Sciences, Isfahan, Iran. *; f *Department of Medical Chemistry, Phytochemistry Research Center, School of Pharmacy, Shahid Beheshti University of Medical Science, Tehran, Iran. *

**Keywords:** Prostate cancer, Alkaloids, Delphinium, cytotoxicity, Apoptosis, mitochondrial pathway

## Abstract

Prostate cancer is one of the common cancers with a high mortality rate in men. Therefore, there is always a necessity to discover new medications for treatment or alleviating its symptoms. In recent years, anticancer properties of a number of delphinium species were reported, but there is no study on the anticancer effects of *Delphinium semibarbatum* (*D. semibarbatum*) alkaloid contents. Therefore, this survey aimed to check the cytotoxicity and apoptotic properties of *D.* *semibarbatum* alkaloid fractions (DSAFs) against prostate cancer cells. Cytotoxicity was measured by MTT assay. We examined the apoptosis by detecting annexin V-FITC/PI staining, the mitochondrial membrane potential (ΔΨm) disruption, reactive oxygen species (ROS) generation, the activity of caspase-3, and expression of the Bax and Bcl-2 in cancer cells. DSAFs treatment inhibited the growth of LNCaP and DU‐145 cells by the increase of apoptotic (Q2+Q3) cells detected by annexin V/PI assay. We found over-generation of intracellular ROS and ΔΨm loss in mitochondrial membrane potential treated cell lines. Attenuation of anti-apoptotic Bcl-2 followed by the increase in pro-apoptotic Bax bands, as well as activation of the caspase-3 enzyme was shown in Western blot analysis. Phytochemical analysis suggested that hetisine type diterpene alkaloids were probably responsible for apoptotic activities. Conclusively, the present study demonstrated that *D.* *semibarbatum* alkaloid content exerted antiproliferative effects against prostate cancer cells by inducing the intrinsic pathway of apoptosis.

## Introduction

Prostate gland cancer is considered one of the common cancers with a high mortality rate in men (1, 2). A large number of patients suffer from drug resistance or resistance to their primary hormonal treatment (2). Therefore, there is always a necessity to investigate new methods for its treatment or to discover new herbal medications for alleviating cancer symptoms. In recent years, *Taxus brevifolia*, *Catharanthus* *roseus* and *Combretum* *caffrum* were successfully studied to discover biomolecules in the treatment of anti-cancer drugs (3). These plants showed prominent anti-tumor properties mediated by different cellular pathways to induce cancer cell apoptosis or proliferation arrest (3, 4).

The genus *Delphinium* sp. (larkspur) belongs to the family Ranunculaceae which is rich in complex diterpene or norditerpene alkaloids with a variant range of toxicity (5). Plant of this genus has limited application as herbal medicines in the treatment of inflammatory disorders like jaundice, rheumatism, toothache, neuralgia (5), and cancer treatment (6). In recent years, a different class of compounds including flavonols, anthocyanins, and alkaloids were isolated from these species from which diterpene alkaloids were more interesting to discover anti-cancer agents (5, 7-9). Interestingly, several reports on the antiproliferative properties of the diterpenoid alkaloids isolated from *Delphinium* species against cancer cells have appeared in recent years. Progress in this field was started in 2010 by Gao`s group, which reported significant cytotoxic activity of Atisine-type alkaloids from *D. chrysotrichum* against the A549 cell line (10). In 2011, C-20 alkaloids isolated from *D. honanense* were reported with potent cytotoxic activity against the breast cancer cells (11). In 2014, hetisine-type C20-diterpenoid alkaloids isolated from *D. Trichophorum* showed prominent cytotoxic activities against A549 cancer cells (12). Therefore, in continuation of recent studies in cancer drug discovery on *Delphinium* species, we selected *D. semibarbatum* to check the cytotoxicity and apoptosis properties of alkaloid fractions of this plant against prostate cancer cells. In Iran, *D. semibarbatum* knowns as yellow larkspur grows wild in the northeast part of the country. *D. semibarbatum* was previously known as a synonym of *D. zalil*, but Sharifnia et al. introduced it as a new species (13). Although anticancer and pharmacological properties of a number of the delphinium species are reported, there is no study on the anticancer effects of *D. semibarbatum* alkaloid contents. 

## Experimental

*Materials*

We purchased JC-1 dye from Sigma Aldrich (St. Louis, MO, USA), an apoptosis kit from Abcam (Cambridge, United Kingdom), and a fluorescent ROS detection kit (Marker GeneTM live cell) from Marker Gene Technologies (Marker Gene Technologies, Inc. Eugene, USA). A colorimetric assay kit for analyses of the caspase-3 enzyme was purchased from an R&D systems corporation (Minneapolis, USA). Sc‐7480 (anti‐Bax), sc‐7382 (anti‐Bcl2), and sc‐47724 as GADPH antibodies were provided from Santa Cruz Biotechnology (CA, USA). DMEM and FBS were purchased from Gibco Invitrogen (Grand Island, NY, USA).

*Plant Material*

*D. semibarbatum *flowering aerial parts were collected in the proximity of Mozdooran Mountains, located between Mashhad and Sarakhs cities, North Khorasan Province, Iran. It was determined by Mohammad Reza Jouharchi, Mashhad University of Sciences, and the voucher specimen (SAM-3392) was stored in the Samsam Shariat Herbarium, Isfahan University of Medical Science. 

*Extraction and Isolation*

Plant material (5000 g) was air-dried at room temperature, powdered, and extracted by ethanol 95% containing 1% trifluoroacetic acid (TFA) with constant shaking for three days and by three times. The acidified ethanol extract was filtered, concentrated by a rotary evaporator, and stored in the refrigerator. To remove fats and chlorophylls, the concentrated extract was filtered through a C-18 cartridge, using ethanol: water: TFA (70:29:1) as solvent. The defatted extract was dried and submitted on a silica gel column using chloroform: methanol (100:0; 95:5; 90:10: 80:20; 70:30; 60:40) as the eluting solvent. Fractions were checked on TLC for alkaloid content using Dragendorff’s reagent. Alkaloid fractions (DSAFs) labeled as Da, Ea, and Eb, and were selected for biological tests against prostate cancer cells.

*Cell Culture*

We prepared DU‐145 and LNCaP (C428 and C439) human prostate cancer cells from the Pasteur Institute of Iran. Cells were cultured at DMEM medium contained 10% FBS, penicillin + streptomycin (100 U/mL+ 100 μg/mL), and maintained in the incubator (37 °C, 5% CO_2_, and 95% humidity).

*Cell Viability Assay*

DU‐145 and LNCaP prostate cancer cells (5000 cells in each well) were seeded in a 96-well plate. After overnight incubation at 37 °C, they were treated with alkaloid fractions in different concentrations of 0.1, 1, 10, 100, 250, 500, and 1000 µg/mL, while the vehicle for each concentration was used as the negative control. After 48 h, MTT solution (0.5 mg/mL, 20 µL) was added to the wells, shacked, and were incubated again at 37 °C, for 4 more h. Then, the supernatants were discarded, DMSO (100 µL) was added, and the optical density was determined at 570 nm by the BioTek microplate reader (Winooski, VT, USA) (14, 15).

*Flow Cytometry Assay with an Apoptosis Kit*

Flow cytometry assay was done as mentioned previously (16). Briefly, we seeded DU‐145 and LNCaP cells (5000 cells/well) in a 6-well plate and incubated them overnight. It was treated with DSAFs (10, 100, and 1000 µg/ml) for 48 h, stained with annexin V-FITC (5 µL) and PI (50 ng/mL, 1 µL), and incubated for 15 min in the dark. Finally, plates were analyzed by Bioscience FACS Calibur flow cytometer (Franklin Lakes, NJ, USA).

*Western Blot Analysis of Apoptotic Related Proteins*

The expression of Bax and Bcl-2 was detected by western blot analysis as reported previously (16). DU‐145 and LNCaP cancer cells were seeded (5000 cells in each well) into the 6-well plates and treated with alkaloid fractions (10, 100, and 1000 µg/ml) for two days. After 48 h, we lysed cells with R0278 RIPA buﬀer containing P7626 PMSF (0.5 mM) and 0.5% P8340 protease inhibitor cocktails (Sigma Aldrich, MO, USA). Then, protein contents of tested samples were determined by the Bradford reagent and equal amounts of proteins (30–50 μg) were resolved by 12% SDS-PAGE and transferred to PVDF membranes (Amersham Pharmacia Biotech). The membranes were incubated with indicated primary antibodies at 4 °C overnight (Bcl-2, Bax, and GADPH, each 1:1000 dilution) followed by another incubation with horseradish peroxidase HRP-conjugated antibodies at room temperature for 2 h. Protein blots were developed by an ECL detection reagent (Amersham Pharmacia Biotech). Finally, each of the western blot bands was analyzed by image j and normalized based on the GAPDH.

*Caspase Activity Assay*

Caspase activity was done as it was reported before (16). Briefly, cells were cultured overnight and treated with DSAFs concentrations (1-1000 µg/mL) for 48 h. Then, we lysed the treated cells, collected the supernatants, and checked them for protease activity. Protease activity was checked by the caspase-specific substrate peptide conjugated with p-nitroanaline as the color reporter. After 1-hour incubation at 37 °C, the amount of released p-nitroaniline was determined at 405 nm.

*Mitochondrial Membrane Potential Assay*

The potential of the mitochondrial membrane was detected using the JC-1 ﬂuorescent probe, which can enter the mitochondria matrix based on the level of ΔΨm. We treated the cells (5 × 10^3^ cells/well) with DSAFs at the concentrations of 1, 10, 100, 500, and 1000 µg/mL. After 48 h, supernatants were discarded and the media were replaced with HEPES buffer (40 mM, pH 7.4) containing JC-1 (2.5 mM) for 30 min at 37 °C. In the end, the fluorescence intensity of the wells was detected at two levels of excitation (490/540 nm)/emission (540/590 nm) wavelengths using BioTek fluorescence Microplate Reader (VT, USA). The difference between 590 and 540 nm fluorescence readings was reported as ΔΨm (17).

*Intracellular ROS Generation Assay*

Generation of reactive oxygen species was analyzed with dichlorofluorescein diacetates (DCFH-DA) as fluorescent probes, which were described previously (17). DU‐145 and LNCaP cells were seeded in a black 384-well plate and treated with DSAFs at concentrations of 1, 10, 100, 500, and 1000 µg/mL for 48 h. Subsequently, they were incubated with DCFH-DA (20 µM) in HEPES (N-2-hydroxyethylpiperazine-N’-2-ethanesulfonic acid) buffer (40 mmol/L, pH 7.4) for 30 minutes at 37 °C in the dark place, and washed with HEPES buffer. The fluorescence intensity was determined at both the excitation and the emission wavelengths of 485 and 528 nm.

*Statistical Analyses*

Results were expressed as mean ± SD. Statistical analysis was done by one-way ANOVA analysis using graph pad software followed by Dunnett’s *post-hoc* test and* P*-value < 0.05 was considered a statistically significant difference.

## Results

*DSAFs Inhibit Cell Proliferation of Prostate Cancer Cell Lines*

**Figure 1:**
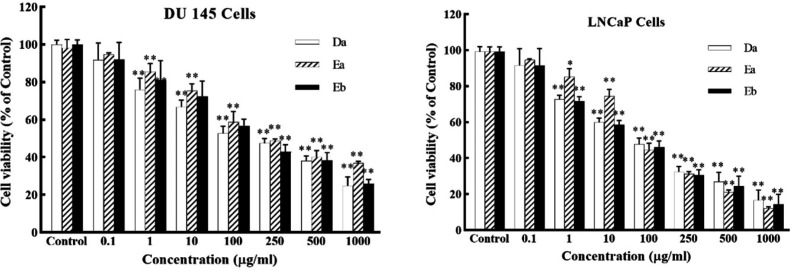
The growth inhibitory effect of *DSAFs on* prostate cancer cells. DU‐145 and LNCaP cells were incubated with different concentrations of *D.* *semibarbatum* alkaloid fractions: Da, Ea, Eb (0.1-1000 µg/**mL**) for 48 h, and cell viability was assessed by MTT assay. Data are presented as the mean ± SD (n = 3). ^*^P < 0.05 and ^**^P < 0.01 *vs.* control group

The inhibitory effect of *D.* *semibarbatum* alkaloid fractions on the growth of prostate cancer cells was primarily evaluated by MTT assay. As shown in Figure 1, DSAFs treatment for 48 h potently suppressed prostate cancer cell growth, both in DU‐145 and LNCaP cells (*P* < 0.05). All the tested fractions inhibited the growth of DU‐145/LNCaP cells in a dose-dependent manner with IC_50_ values of Da: 62.33 ± 2.52/82.50 ± 3.53; Ea: 173.33 ± 15.27/75.80 ± 13.54; Eb: 98.33 ± 15.57/89.6 ± 6.22 varied from 62.33 –173.33/75.80 - 98.33 μg/mL, respectively. In comparison between fractions, Da exhibited higher cytotoxic effects in DU 145, and Ea in LNCaP treated cells (Figure 1).

*DSAFs Induce Apoptosis* *in Prostate Cancer Cell Lines*

**Figure 2:**
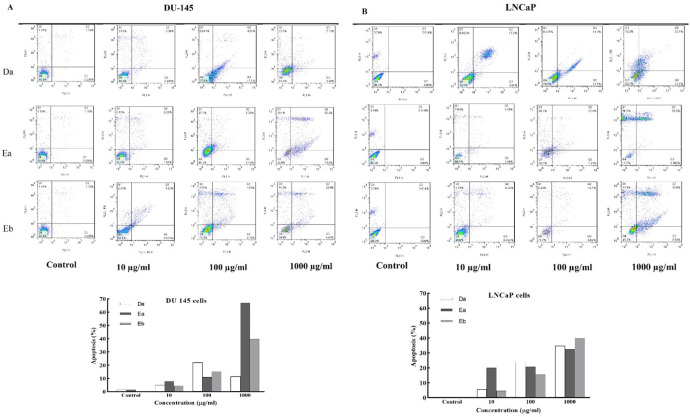
*DSAFs induced* cell death by apoptosis in prostate cancer cells. Cells were treated with various concentrations of *D.* *semibarbatum* fractions (10, 100 and 1000 µg/**mL**) for 48 h, and **then** annexin V/PI-stained cells were analyzed **using flow cytometry. The results were** **shown as flow cytometry charts in **DU‐145 (**A) **and LNCaP **(**B**) **cells. Data are presented as the mean ± SD (n = 3). ^*^P < 0.05 and ^**^P < 0.01 *vs.* control group

We stained the treated cells by annexin V/PI and analyzed them by flow cytometry to determine the type of cell death. Staining was conducted to classify cells following treatment with DSAFs into three groups: viable (double negative stained; Q1), apoptotic (Annexin V-FITC^+^; Q2+Q3), and necrotic (Annexin V-FITC^-^/PI^+^; Q4) cells. As it can be seen in Figure 2, alkaloid fractions significantly increased the number of apoptotic cells, compared to the untreated cells in a dose-dependent manner (*P* < 0.05). Specifically, cell apoptosis increased in the range of 4.95 – 11.46% (Da), 8.01 – 67% (Ea), and 11.84 – 27.43% (Eb) for DU‐145 cells (Figure 2A) and 5.65 – 34.2% (Da), 20.05 – 32.36% (Ea), and 4.64 – 39.83% (Eb) for LNCaP cells (Figure 2B). At a higher concentration (1000 μg/mL), the highest apoptogenic activity was observed for fraction Ea against DU‐145 (67%). Other than apoptosis, necrotic death was also seen; but, the predominating cell death by DSAFs was through the increased population of apoptotic cells (Annexin V-positive) in a dose-dependent manner, especially in DU 145 cells. 

*Caspase-3 Activity in DSAFs Treated with DU‐145 and LNCaP Cells*

**Figure 3:**
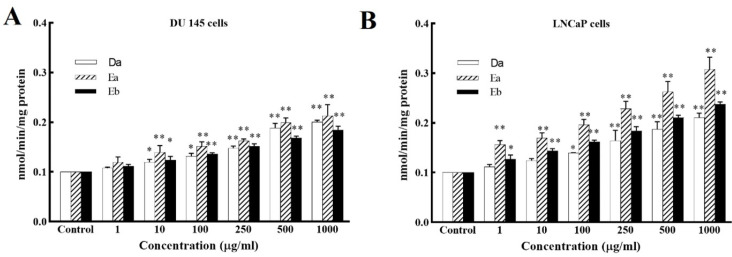
*DSAFs *induced apoptosis through caspase-3 activation in prostate cancer cells. Colorimetric assay of caspase-3 activation after treatment with various concentrations of *D.* *semibarbatum* fractions was measured in DU‐145 and LNCaP cells. Values are presented as means ± SD (n = 3). ^*^P < 0.05 and ^**^P < 0.01 *vs.* control group

To examine the downstream effectors in the apoptotic signaling pathway, activation of caspase-3 was examined by a colorimetric assay using specific chromophores. As shown in Figure 3, *D.* *semibarbatum* alkaloid fractions significantly, and dose-dependently enhanced caspase-3 activity in both cell lines (*P* < 0.05). According to these results, the Ea fraction showed more activity on caspase-3 than others. These data indicated that DSAFs induced apoptosis through the caspase pathway in both prostate cancer cells.

*DSAF (Ea) Induces Depolarization of Mitochondrial Membrane Potential (ΔΨm)*

**Figure 4:**
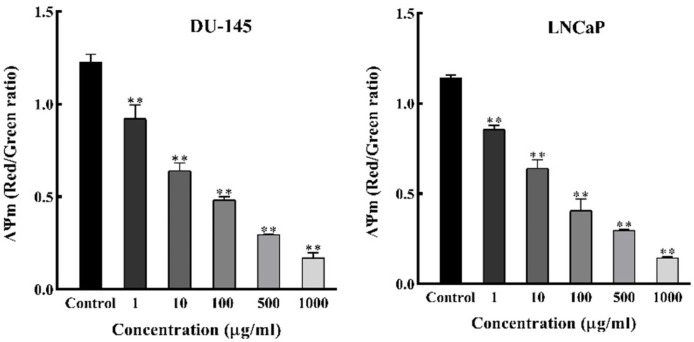
Depolarization of mitochondrial membrane potential in prostate cancer cells. After treatment with increasing concentrations of Ea (selected alkaloid fraction of D. semibarbatum) for 48 h, cells were loaded with JC-1 dye and the ΔΨm was directly measured. Values are presented as means ± SD (n = 3). *P < 0.05 and **P < 0.01 vs. control group

As it is known, loss of ΔΨm is an important event in cell apoptosis. For further assessment on how apoptosis occurs in *DSAFs treated* cells, the mitochondrial membrane potential was analyzed by DSAF (Ea) as a selected fraction by employing a JC-1 fluorescent probe. As shown in Figure 4, a significant loss of ΔΨm occurred after treatment with various concentrations of Ea (1-1000 µg/mL) in a dose-dependent manner in DU‐145 and LNCaP cells (*P* < 0.05). These results confirmed that DSAF (Ea) induced apoptosis on DU‐145 and LNCaP cells after 48 h through the disruption of the mitochondrial membrane.

*DSAF (Ea) Increases ROS Generation*

**Figure 5:**
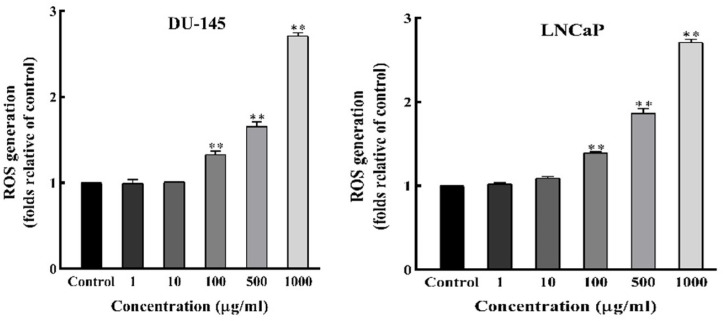
ROS generation in prostate cancer cells. The level of ROS was detected by DCFH-DA after treatment with DSAF (Ea) (1-1000 µg/ml) for 48 h in DU‐145 and LNCaP cells. Values are presented as means ± SD (n = 3). *P < 0.05 and **P < 0.01 vs. control group

To better characterize the pathway through which *D.* *semibarbatum* fractions exert apoptosis in prostate cancer cells, the impact of one of the fractions: Ea of *D.* *semibarbatum* on intracellular ROS generation was assessed with the specific DCFH-DA probe. After treatment with Ea for 48 h, a significant increase in ROS levels was observed in a dose‐dependent manner, starting from 50 µg/mL (*P* < 0.05, Figure 5). These data indicated that ROS overproduction is involved in induced apoptosis by fraction Ea.

*Western Blot Analysis of the Anti-apoptotic and Pro-Apoptotic Proteins in DU‐145 and LNCaP Cells*

**Figure 6:**
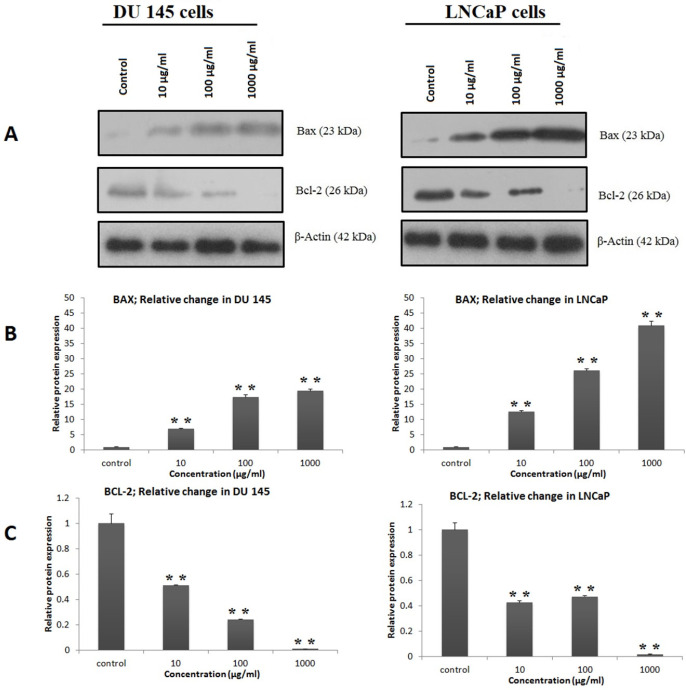
DSAF (Ea) regulated anti-apoptotic and pro-apoptotic proteins in DU‐145 and LNCaP prostate cancer cells. DU‐145 (A) and LNCaP (B) cells were treated with the indicated concentrations of DSAF (Ea) for 48 h and then the expression levels of proteins were assayed by western blotting and corresponding antibodies

Western blot analysis was employed to determine the effect of fraction Ea from *D.* *semibarbatum* on the expression of proteins involved in the mitochondria-mediated apoptosis. As shown in Figure 6, after 48 hof Ea treatment, the expression of Bcl-2 was downregulated, while the Bax was upregulated (*P* < 0.05). These results confirmed that Ea treatment induces apoptosis in DU‐145 and LNCaP cells via the activation of the mitochondrial pathway. 

## Discussion

This study examined the antiproliferative effect of *D. semibarbatum* against prostate cancer cells. The MTT assay showed significant antiproliferative effects against LNCaP and DU‐145 cells with higher suppressive activity against LNCaP cells. 

Many of the anti-cancer agents can induce apoptosis (18). Since apoptosis is the most potent defense in multicellular organisms against cancer phenomena, the selective induction of apoptosis in tumor cells is a promising therapy approach (19, 20). Here, we demonstrated that compared to the untreated cells, DSAFs decreased cell viability through apoptosis in both LNCaP and DU‐145 prostate cancer cells. Apoptosis was confirmed by an increased population of the annexin positive stained cells in flow cytometry analysis using annexin V/PI. Our results indicated that DSAFs increased both early and late-stage apoptosis in a dose-dependent manner, with high apoptogenic activity at the higher concentrations.

Mitochondria’s role is important in regulating the pathways leading to apoptosis (21). Figure 5 clearly showed that DSAFs induced apoptosis concomitant with loss of ΔΨm in both cell lines. It indicated that anti-cancer activity is exerted by mitochondrial membrane depolarization, and possibly is related to the elevation of ROS levels (22). The inhibitory effects of some anti-tumor drugs are dependent on ROS generation (23). In the mitochondrial-dependent cell apoptosis, excessive ROS production can distract the mitochondria membrane, release apoptosis-inducing factors, and start the caspase cascades (22). Consistent with these results, and intracellular ROS overproduction, we proposed that DSAFs induce apoptosis via the mitochondria-dependent pathway by an increase in intracellular ROS and loss of ΔΨm, which consequently leads to a cascade of events driving to apoptosis (21).

In the mitochondrial pathway of apoptosis, relevant studies confirmed that a high Bax to Bcl-2 ratio is associated with the ΔΨm decrease following cytochrome c release (21). The facilitated release of cytochrome c from mitochondria into the cytosol, as mentioned before, induces caspase cascades which led to apoptosis (24). Therefore, we analyzed the expression of the Bax/ Bcl-2 ratio by western blot analysis and showed that DSAFs treatment shifts the Bax/Bcl-2 ratio in favor of apoptosis by a significant decrease in the anti-apoptotic Bcl-2, and increase of the pro-apoptotic Bax protein (Figure 4). The excess activation of the Bax/Bcl-2 ratio increases permeabilization of the mitochondrial outer membrane and consequently causes the release of other pro-apoptotic molecules, which induce apoptosis through caspase cascades (24). It was consistent with the caspase-3 activity results significantly increased by DSAFs in a dose-dependent manner (Figure 3).

Phytochemical analysis of DSAF (Ea) led to the isolation of one unprecedented C-20 hetisine type diterpene alkaloid as the major component which is reported elsewhere. In this agreement, Spiramine C–D from the hetisine-type diterpenoid alkaloids showed apoptosis activity in MCF-7 cells (25). Xiaoxia Liang *et al.* reported similar diterpene alkaloids with anti-cancer activities (26). Hazawa et al. reported that six hetisine-type derivatives showed significant cytotoxicity against the Raji and A549 cells (27). In another research surveyed by Xiangting Gao, aconitine with (C-19 diterpene alkaloid) was tested against H9c2 cardiac cells. Aconitine induced intrinsic apoptosis by upregulation of cytochrome c, Bax/Bcl-2 ratio, and caspase-3 activity (28).

## Conclusion

Conclusively, the present study demonstrated that all the three alkaloidal fractions of *D. semibarbatum* exert cytotoxic activities, through apoptosis against LNCaP, and DU 145 cancer cells. Although other than apoptosis, necrotic death was also seen; but, the predominating cell death by DSAFs was through the increased population of apoptotic cells, especially in DU 145 cells. The apoptosis mechanism was investigated to be through the intrinsic pathway. The induction of apoptosis was found to be associated with increased ROS generation, disruption of mitochondrial membrane potential (MMP), and activation of caspase-3. Western blot analysis confirmed that treatment with DASF (Ea) in DU‐145 and LNCaP cells increases the Bax/Bcl-2 ratio through the release of BAX pro-apoptotic, and decreasing Bcl-2 anti-apoptotic proteins.
